# Case series: psoriasis in solid organ transplant patients and immunobiological agents^☆^

**DOI:** 10.1016/j.abd.2022.11.004

**Published:** 2023-05-16

**Authors:** Luana Pizarro Meneghello, Diéssica Gisele Schulz, Larissa Prokopp da Costa, André Vicente Esteves de Carvalho

**Affiliations:** a Department of Dermatology, Universidade Franciscana, Santa Maria, RS, Brazil; b Department of Dermatology, Hospital Moinhos de Vento de Porto Alegre, Porto Alegre, RS, Brazil

Dear Editor,

Psoriasis treatment scenario has progressed rapidly over the last few decades; however, the use of immunobiologicals in immunocompromised individuals with psoriasis is rarely discussed in the literature.^1^ In transplant recipients, the potential risks of combining immunosuppressants with biological agents for the treatment of psoriasis are not fully known, as these patients are usually excluded from pivotal clinical trials.^2^

Immunocompromised patients, such as organ transplant recipients, require a careful risk-benefit assessment when selecting an immunobiological for the treatment of psoriasis.^1^ Due to the degree of immunosuppression necessary to prevent graft rejection, the acute form of the disease tends to disappear in the immediate period after the procedure. However, during the transition phase to a maintenance dose of immunosuppression, psoriasis may exacerbate. The transplant recipient body response can be influenced by the choice of immunosuppressive therapy, the transplanted organ, and individual tolerability.^2^

Currently, there are no guidelines for the treatment of moderate to severe psoriasis in transplant patients, and only a few case reports on the use of biologicals together with an immunosuppressive regimen have been described in the literature (Table 1).^3^ The authors report three cases of transplant patients receiving immunobiological agents for the treatment of psoriasis.

**Table 1:** Literature review on patients with psoriasis and previous solid organ transplantation who received immunobiological therapy

Author	Age, sex	Transplanted organ	Immunosuppressive regimen	Immunobiological	Time of use	Clinical response	Complications
Present study	53, M	Kidney	Sirolimus and Acitretin	Ustekinumab	6 years	Yes	None
Present study	45, M	Liver	Tacrolimus and Mycophenolate Mofetil	Etanercept	‒	Yes	None
Present study	39, M	Kidney	Tacrolimus and Prednisone	Risankizumab	2021 to date	Yes	None
Blasco et al., 2022^10^	29, H	Kidney	Prednisolone and Tacrolimus	Adalimumab	‒	Yes	None
Singh et al., 2021^1^	50, H	Liver	Tacrolimus and Mycophenolate Mofetil	Brodalumab	‒	Yes	None
Richetta et al., 2021^7^	50, H	Liver	Tacrolimus and Mycophenolate Mofetil	Ustekinumab	6 months	Yes	None
Lora et al., 2019^9^	54, H	Liver	Tacrolimus and Mycophenolate Mofetil	Ixekizumab	2 years	Yes	None
Madankumar et al., 2015^6^	52, M	Liver	Tacrolimus, Prednisone and Everolimus	Etanercept	1 year	Yes	Multiple infections*
Brokalaki et al., 2012^5^	42, M	Pancreas-kidney	Prednisone, Tacrolimus and Mycophenolate Mofetil	Etanercept	2 years	Yes	Not reported
Hoover et al., 2007^a^	63, M	Liver	Tacrolimus and Sirolimus	Etanercept	6 months	Yes	None
Collazo et al., 2008^b^	49, M	Liver	Tacrolimus, Mycophenolate Mofetil and Corticosteroids	Etanercept	5 months	Yes	None
García-Zamora et al., 2018^c^	67, H	Kidney	Tacrolimus, Mycophenolate Mofetil and Corticosteroids	Etanercept	10 months	Yes	None
De Simone et al., 2016^d^	56, H	Liver	Prednisone, Tacrolimus and Mycophenolate Mofetil	Etanercept	6 months – re-starting after recurrence	Yes	None

M, Male; F, Female; ‒, No data.

a Hoover WD. Etanercept therapy for severe plaque psoriasis in a patient who underwent a liver transplant. Cutis. 2007;80:211-214.

b Collazo MH, González JR, Torres EA. Etanercept therapy for psoriasis in a patient with concomitant hepatitis C and liver transplant. P R Health Sci J. 2008;27:346-347.

c García-Zamora E, Gómez de la Fuente E, Miñano-Medrano R, López-Estebaranz JL. Uso de etanercept en un paciente trasplantado renal con psoriasis. Actas Dermosifiliogr. 2020;111:169-171.

d De Simone C, Perino F, Caldarola G, D'Agostino M, Peris K. Treatment of psoriasis with etanercept in immunocompromised patients: Two case reports. J Int Med Res. 2016;44:67-71.

* Asymptomatic urinary tract infections and recurrent cholangitis.

## Case I

A 53 years old male was a kidney transplant recipient due to diabetic glomerulopathy. He had psoriasis since childhood and type II diabetes and used methotrexate in the period prior to kidney transplantation. The post-transplant immunosuppressive regimen initially included azathioprine, prednisone, and cyclosporine for five years, achieving psoriasis control. During this time, the patient developed several viral, disseminated verrucous lesions. Neoplastic transformation to moderately differentiated invasive squamous cell carcinoma occurred in the lesions located on the face and perineum. He underwent surgical treatment with frozen section control. Due to the possibility of the development of other lesions, the immunosuppressive regimen was changed to sirolimus. However, psoriasis recurred, with a Psoriasis Area and Severity Index (PASI) score of 39.4; Body Surface Area (BSA) of 48, and Dermatological Life Quality Index (DQLI) of 18. On that occasion, acitretin 35 mg/day was added to further decrease the verrucous lesions and control psoriasis, which showed some pustular lesions. There was no positive response after three months of follow-up and, in agreement with the Nephrology team, ustekinumab was started at a dose of 90 mg at weeks 0 and 4 then every 12 weeks, maintaining the use of sirolimus and acitretin at a dose of 10 mg/day. After eight weeks of treatment, he had a PASI score of 5.0; BSA of 12, and DLQI of 1. Psoriasis had been stable for four years when the patient described in this case was diagnosed with a metastatic tumor in the lung and died.

## Case II

**Figure 1:**
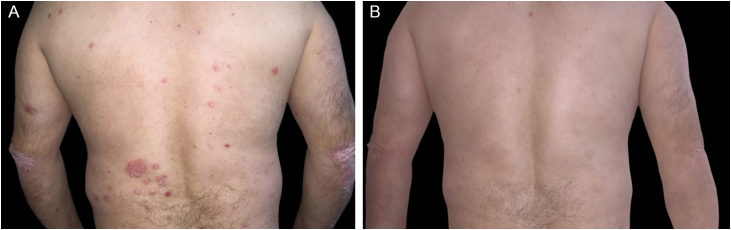
(A) Scaling erythematous plaques on the back and elbows. (B) Complete resolution after treatment with etanercept

A 45 years old male was a liver transplant recipient due to autoimmune hepatitis. He was diagnosed with plaque psoriasis, with pre-transplant PASI of 12, BSA of 16, and DLQI of 11. He was treated with topical medications and phototherapy. After the transplant and having started immunosuppressive therapy with corticosteroids and calcineurin inhibitor, the lesions showed complete improvement, until the regimen was interrupted and replaced by tacrolimus and mycophenolate mofetil six months later. He showed worsening of the lesions with a PASI score of 21.4; BSA of 29 and DLQI of 18. Treatment with phototherapy without psoralen was started but it was suspended after three sessions due to intense erythema and burns. After discussion with the Gastroenterology and Transplantation Medicine teams, etanercept was started at a dose of 50 mg per week, with significant improvement in the patient condition (PASI of 2.2; BSA of 08, DLQI of 0) in the twelfth week of medication use (Fig. 1). The patient has remained stable.

## Case III

**Figure 2:**
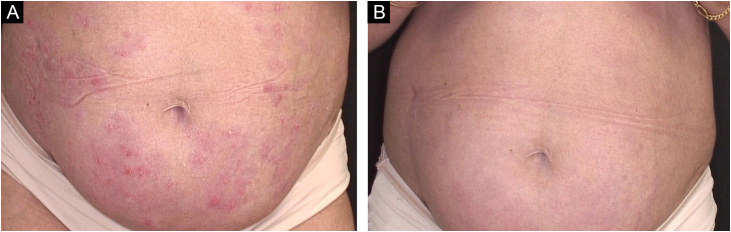
(A) Erythematous plaques on the abdomen. (B) Complete resolution after treatment with risankizumab

A 39 years old female was a kidney transplant recipient due to thrombocytopenic purpura. She was diagnosed with psoriasis at ten years old. She underwent multiple treatments, ranging from topical medications to methotrexate. She used acitretin for 22 years, reaching a dose of 50 mg/day. Due to the effects of the medication, the dose was reduced to 25 mg/day in the last five years without reaching a PASI 90 response. On clinical examination, she had plaque psoriasis, PASI 10.10, BSA of 11% and DLQI of 17. Due to the clinical picture and quality of life impairment in a renal transplant recipient unresponsive to previous therapies, treatment with subcutaneous risankizumab at a dose of 150 mg at weeks 0, 4 and then every 12 weeks was chosen. Sixteen weeks after the first application, the patient had a PASI score of 90 and a DLQI of 0. The patient is currently completing 30 weeks of follow-up, with excellent clinical response and without any complications (Fig. 2).

As seen in the cases described above, immunobiological therapy in immunocompromised patients is used in cases of difficult-to-treat psoriasis. Few studies have been performed on the use of anti-TNF-α therapy in kidney transplant patients. Some cases described in the literature reported that disease improvement has been achieved without adverse effects with the use of etanercept.^4^, ^5^, ^6^, ^7^

This is the second case report to date of psoriasis in transplant recipients treated with ustekinumab with rapid and complete disease resolution without any side effects.^7^ No increased risk of malignancies, severe cardiovascular adverse events, severe infections, or increased mortality was observed in patients with psoriasis while receiving this drug.^8^

To date, Case III of this report is the first case describing the use of risankizumab in organ transplant recipients with psoriasis. The use of risanquizumabe has a good safety profile and a low rate of adverse effects.^8^ Some authors have reported the use of ixekizumab, brodalumab, and adalimumab as safe options in the treatment of transplant patients with severe psoriasis.^1^, ^9^, ^10^

With these new cases, the authors provide additional information on the use of immunobiologicals in transplant recipients. It is critical that the patient, Dermatology team, and Transplantation Medicine team work together to address the challenges and relatively unfamiliar scenarios of psoriasis treatment in transplant recipients. As the number of organ transplants is on the rise, this issue will increasingly become clinically relevant.

## Financial support

None declared.

## Authors' contributions

Luana Pizarro Meneghello: Effective participation in research orientation; intellectual participation in the propaedeutic and/or therapeutic conduct of the studied cases; critical review of the manuscript; approval of the final version of the manuscript.

Diéssica Gisele Schulz: Drafting and editing of the manuscript; critical review of the literature; approval of the final version of the manuscript.

Larissa Prokopp da Costa: Drafting and editing of the manuscript; critical review of the literature; approval of the final version of the manuscript.

André Vicente Esteves de Carvalho: Effective participation in research orientation; intellectual participation in the propaedeutic and/or therapeutic conduct of the studied cases; critical review of the manuscript; approval of the final version of the manuscript.

## Conflicts of interest

None declared.
